# Supplementary material for: A Mechanistic Pharmacodynamic Modeling Framework for the Assessment and Optimization of Proteolysis Targeting Chimeras (PROTACs)
Source: Pharmaceutics. 2023 Jan 5;15(1):195. doi: 10.3390/pharmaceutics15010195 (PMC9865105; doi:10.3390/pharmaceutics15010195)
Supplement: Supplementary file 1 [file pharmaceutics-15-00195-s001.zip › Script_Hook_Model.pdf]

# 
********************************************************************************
******************************************************

# Header:

# Script for fitting data on PROTAC-mediated Protein Of Interest degradation to drug exposure

# Version: 1.0 (November 10th, 2022)

# Author: Robin T.U.HAID

# R version: 4.1.2 (2021-11-01)

# Required packages: None

# 
********************************************************************************
******************************************************

# Nomenclature (dimensions/units in brackets):

# P ... relative levels of protein of interest remaining (e.g. %)

# C ... constant drug concentration used in the assay (e.g. nM)

# Dmax ... maximum extent of degradation that can be observed (e.g. %)

# DCmax ... drug concentration where Dmax is observed (e.g. nM)

# DC50 ... drug concentration where Dmax/2 is observed (e.g. nM)

# P0_cor ... correction coefficient to account for imprecise DMSO control (e.g. %)

#       P0_cor adjusts the reference P levels (i.e. the first plateau)

# n ... empirical Hill coefficient that gives the steepness of the curve in the Emax model (e.g. 1)

# 
********************************************************************************
******************************************************

# Functions:

# The first of the fours functions (i.e. "Emax") is the conventional Emax model, which is used when there is no "hook" effect present in the data (NOT new)

#    NOTE: the effect (E) of interest is degradation (D)

#    NOTE: for DC50 the log of DC50 (i.e. log_DC50) is fitted, in order to avoid negative concentrations (only relevant for standard error)

# The second function (i.e. "Hook") contains the expression for fitting the "hook" model to experimental data,

RESTRICTED

```
#   it can be used with e.g. the nls function (as the "formula" argument)
#     NOTE: the "Hook" function is about degradation (D) & NOT P remaining (P), with D:=P0-P where P0 is either 1 or 100%
#     NOTE: for DC50 & DCmax the logs of DC50 & DCmax (i.e. log_DC50 & log_DCmax) are fitted, in order to avoid negative concentrations (only relevant for standard error)
# The third function ("Hook_fit") takes two vectors (one for P levels remaining (P) & one for PROTAC conc. (C)) and returns
#   a fitted model (fit performed according to "Hook" function or the "Emax" model respectively) assuming that the residual error follows a
#   log-normal distribution
#     NOTE: The function fits the log of P levels and therefore also predicts the respective log values
#           (to transform back to linear scale use: exp(predict(model)), where "model" is the name of the fitted model)
#     NOTE: The function can make reasonable guesses for the initial parameter estimates &
#           P can be either given as a fraction (e.g. 0.5 instead of 50%) or in percent (%), while C can be any concentration (e.g. nM or M)
# The fourth function ("Hook_analyse") also returns the fitted model but additionally also generates an overlay-plot
#     NOTE: The "Hook_analyse" function only works with a data.frame as input,
#           where P levels and PROTAC concentrations must be named "P" and "C" respectively
#     NOTE: The data.frame must also contain the "ID" (i.e. name) of the dose-response profile as well as the "unit" of the conc.


# 0)
Emax <- function(Dmax, log_DC50, n, C){ # describes degradation as a function of drug concentration (no "hook" effect -> conventional Emax model)
  DC50 <- exp(log_DC50) # convert log parameter to actual parameter
  D <- Dmax * (C^n/(C^n+DC50^n)) # degradation (D:=1-P)
  return (D) # returns degradation, NOT P levels remaining (P=1-D)
} # end of function "Emax"


# 1)
Hook <- function(Dmax, log_DC50, log_DCmax, C){ # describes degradation as a function of drug concentration (accounting for the "hook effect" -> name)
```

RESTRICTED

```
  DC50 <- exp(log_DC50) # convert log parameter to actual parameter

  DCmax <- exp(log_DCmax) # convert log parameter to actual parameter

  D <- Dmax * (DC50+DCmax^2/DC50-2*DCmax) / (DC50+DCmax^2/DC50-4*DCmax+C+DCmax^2/C) #
degradation (D:=1-P)

  return (D) # returns degradation, NOT P levels remaining (P=1-D)

} # end of function "Hook"



# 2)

Hook_fit <- function(P, C){ # fits data on P degradation to data on drug exposure -> returns an nls
object

  P <- P[!is.na(P)&!is.na(C)] # makes sure that there are no missing values

  C <- C[!is.na(P)&!is.na(C)] # makes sure that there are no missing values

  if (max(P)>2){ # checks whether P is given in percent (%)

    P0 <- 100 # P0 (P levels in the absence of drug exposure) is set to 100 %

  } else { # if P is not given in percent (%) then it must be given as a fraction (e.g. 0.5 instead of 50%)

    P0 <- 1 # P0 (P levels in the absence of drug exposure) is set to 1

  } # end of code determining the unit of P

  Dmax_estimate <- mean(P0-min(P)) # estimate Dmax to obtain a starting value for fitting

  DCmax_estimate <- mean(C[P==min(P)]) # estimate DCmax to obtain a starting value for fitting

  DC50_estimate <- min(C[(P[C<DCmax_estimate]-(P0-
(Dmax_estimate/2)))^2==min((P[C<DCmax_estimate]-(P0-(Dmax_estimate/2)))^2)]) # estimate DC50
to obtain a starting value for fitting


  if (sum(P[C>=DCmax_estimate])/length(which(C>=DCmax_estimate))>(P0-Dmax_estimate)*1.05){ #
checks, whether there is a "hook" effect

    Hook_model <- nls(log(P)~log(P0_cor*(P0-Hook(Dmax, log_DC50, log_DCmax, C))), # the "Hook"
function is about degradation & NOT about P levels remaining

                    start=c(Dmax=Dmax_estimate, log_DC50=log(DC50_estimate),
log_DCmax=log(DCmax_estimate), P0_cor=1),

                    algorithm="port", lower=c(0, -Inf, -Inf, 0), upper=c(P0, Inf, Inf, Inf),
control=c(warnOnly=TRUE)) # estimates for start values

    return (Hook_model) # returns the fitted model (nls object)

  } else { # if there is no "hook" effect, then the conventional (Emax) model is fitted

    Emax_model <- nls(log(P)~log(P0_cor*(P0-Emax(Dmax, log_DC50, n, C))),
```

RESTRICTED

```
                    start=c(Dmax=Dmax_estimate, log_DC50=log(DC50_estimate), n=1, P0_cor=1),
                    algorithm="port", lower=c(0, -Inf, -Inf, 0), upper=c(P0, Inf, Inf, Inf),
control=c(warnOnly=TRUE)) # estimates for start values
    return (Emax_model)
  }
} # end of function "Hook_fit"



# 3)
Hook_analyse <- function(data){ # plots the experimental data together with the fitted model &
returns the fitted model
  ID <- data$ID # extract all the IDs (compound identifier)
  idx <- 0 # initialize index (index of model that is fitted)
  for (i in unique(ID)){ # fit one model for each compound
    P <- data[data$ID==i,]$P # extract P levels from data (assuming "data" is a data.frame)
    if (max(P)>2){ # checks whether P is given in percent (%)
      P0 <- 100 # P0 (P levels in the absence of drug exposure) is set to 100 %
    } else { # if P is not given in percent (%) then it must be given as a fraction (e.g. 0.5 instead of 50%)
      P0 <- 1 # P0 (P levels in the absence of drug exposure) is set to 1
    } # end of code determining the unit of P
    C <- data[data$ID==i,]$C # extract PROTAC conc. from data (assuming "data" is a data.frame)
    unit <- unique(data[data$ID==i,]$unit) # extract the unit of the conc. from data (assuming "data" is
a data.frame)
    Hook_model <- Hook_fit(P, C) # calls function "Hook_fit" to fit the "Hook" model to the data
    min_C <- round(min(log10(C)))-1 # log10 of lower limit of x-axis
    max_C <- round(max(log10(C)))+1 # log10 of upper limit of x-axis
    ticks_Conc <- seq(from = min_C, to = max_C) # location of tick-marks on the x-axis
    labels_Conc <- sapply(ticks_Conc, function(i) # labels of the tick-marks (always 10^ticks_Conc since
ticks_Conc is in log10)
      as.expression(bquote(10^ .(i)))) # labels are converted to expressions
    xlab <- bquote("[PROTAC] -"~.(unit)) # to specify label of x-axis in following plot
    plot(las=1, P*100/P0~log10(C), data=data, ylim=c(0, 120), yaxs="i", type="n", # creates an overlay-
plot
```

RESTRICTED

```
        xlim=c(min_C, max_C), xaxs="i", axes=FALSE, # set sensible limits to the x-axis
        xlab=xlab, # the unit for [PROTAC] has to be specified in the input data.frame
        ylab=c("P levels remaining - (%)"), # always in percent (%)
        main=i)
  axis(side=1, at=ticks_Conc, las=1, # add axis
        labels=labels_Conc) # enter tickmark labels (logarithmic axis)
  axis(side=2, at=seq(0, 120, 20), las=1) # add axis
  axis(side=3, at=ticks_Conc, labels=FALSE, tcl=0) #  tcl=0 creates a blank axis without any tick points
  axis(side=3, at=ticks_Conc[-c(1, length(ticks_Conc))], labels=FALSE, tcl=.5, lwd=0, lwd.ticks=1) # tcl=.5 makes tick points face inwards
  axis(side=4, at=seq(0, 120, 20), labels=FALSE, tcl=0, las=1) #  tcl=0 creates a blank axis without any tick points
  axis(side=4, at=seq(20, 100, 20), labels=FALSE, tcl=.5, las=1, lwd=0, lwd.ticks=1) #  tcl=.5 makes tick points face inwards
  if(names(coef(Hook_model)[3])=="log_DCmax"){ # check whether the "hook" model or the Emax model was used
    Dmax <- round(coef(Hook_model)[1]*100/P0) # extract the fitted parameters
    DC50 <- signif(exp(coef(Hook_model)[2]), 2) # extract the fitted parameters
    DCmax <- signif(exp(coef(Hook_model)[3]), 2) # extract the fitted parameters
    P0_cor <- round(100*coef(Hook_model)[4]) # extract the fitted parameters
    legend("bottomleft", inset=c(0.01, 0.0185),
           legend=c(
             bquote(D[max] ~ "=" ~ .(Dmax) * "%"),
             bquote(DC[50] ~ "=" ~ .(DC50) ~ .(unit)),
             bquote(DC[max] ~ "=" ~ .(DCmax) ~ .(unit)),
             bquote(P[0] ~ "=" ~ .(P0_cor) * "%"),
             expression()
           ))
  } else {
    Dmax <- round(coef(Hook_model)[1]*100/P0) # extract the fitted parameters
    DC50 <- signif(exp(coef(Hook_model)[2]), 2) # extract the fitted parameters
    n <- signif(coef(Hook_model)[3], 2) # extract the fitted parameters
    P0_cor <- round(100*coef(Hook_model)[4]) # extract the fitted parameters
```

RESTRICTED

```
    legend("bottomleft", inset=c(0.01, 0.0185),
           legend=c(
             bquote(D[max] ~ "=" ~ .(Dmax) * "%"),
             bquote(DC[50] ~ "=" ~ .(DC50) ~ .(unit)),
             bquote(n ~ "=" ~ .(n)),
             bquote(P[0] ~ "=" ~ .(P0_cor) * "%"),
             expression()
           ))
  }
  points(exp(predict(Hook_model, data.frame("C"=10^seq(min_C, max_C,
0.01))))/P0*100~seq(min_C, max_C, 0.01), type="l") # plot the fitted model (graph)
  points(P*100/P0~log10(C), pch=21, bg="white") # plot the experimental data
  if (idx==0){ # start a new list, if this is the first compound fitted
    r <- (Hook_model)
  } else { # add a new model (for a new compound) to the list of models
    r <- list(r, Hook_model) # add model to vector of all models
  }
  idx <- idx+1 # update index
 }
 names(r) <- unique(ID) # name the models after the compound identifiers
 return(r) # return the fitted models (nls objects)
} # end of function "Hook_analyse"


#
*************************************************************************************
********************************************************
# Application:


# dummy data are taken from: Zorba et al.; Delineating the role of cooperativity in the design of potent PROTACs for BTK. Proc. Natl. Acad. Sci. USA (2018)
# "P" could also be given as fractions (e.g. 0.5 instead of 50%) and "C" could also be in any other unit for concentrations
```

RESTRICTED

```
# the names of the variables must be "C", "P", "ID" & "unit"

data_1 <- data.frame("C"=c(1, 10, 30, 100, 300, 1000, 3000, 10000, 30000), # in nM (experimental
data)

                    "P"=c(114, 93, 87, 74, 55, 35, 31, 44, 68)) # in % (experimental data)

data_1$ID <- "Compound X" # name that is displayed in the output plot

data_1$unit <- "nM" # unit of the concentrations

data_2 <- data.frame("C"=c(1, 10, 30, 100, 300, 1000, 3000, 10000, 30000), # in nM (experimental
data)

                    "P"=c(0.98, 0.98, 1, 1, 0.87, 0.73, 0.67, 0.68, 0.78)) # in 1 (experimental data)

data_2$ID <- "Compound Y" # name that is displayed in the output plot

data_2$unit <- "M" # unit of the concentrations

data <- rbind(data_1, data_2) # combine the individual data.frames (one for each compound) into
one


model <- Hook_analyse(data) # function "Hook_analyse" generates an overlay-plot and returns the
fitted nls model

model$"Compound X" # gives the model for Compound X (if the data frame contains multiple
different compounds,

# then there will be one entry in the list per compound

model[["Compound X"]] # does the same

P_pred <- exp(predict(model$"Compound X")) # returns the predicted P levels for compound X

# NOTE: exp() is necessary, since the model uses a log-normal error model

#       (so that the fit is best at low P levels, where there is a lot of degradation -> more precisely
estimate Dmax & DC50)

DC50_CI <- exp(confint(model[["Compound X"]], level = 0.95))["log_DC50",] # returns the 95%
confidence interval for DC50 for Compound X

# NOTE: exp() is necessary since the model assumes that the estimates of log_DC50 & log_DCmax
follow a normal distribution

#       (this ensures that there will never be a concentration smaller than 0)

# NOTE: it might be that confint() fails if the "level" is too high (then try a smaller level that is a
narrower confidence interval)


# end

#
********************************************************************************
*****************************************************
```

RESTRICTED
