# Supplementary material for: A Mechanistic Pharmacodynamic Modeling Framework for the Assessment and Optimization of Proteolysis Targeting Chimeras (PROTACs)
Source: Pharmaceutics. 2023 Jan 5;15(1):195. doi: 10.3390/pharmaceutics15010195 (PMC9865105; doi:10.3390/pharmaceutics15010195)
Supplement: Supplementary file 1 [file pharmaceutics-15-00195-s001.zip › Supplementary Materials.pdf]

A Mechanistic Pharmacodynamic Modeling Framework for the Assessment and Optimization of Proteolysis Targeting Chimeras (PROTACs)

Robin Thomas Ulrich Haid [1,2] and Andreas Reichel [1,*]

Supplement

1. Figure S1: comparison hook model and $E_{\max}$ model – RAMOS cells
2. Figure S2: comparison hook model and $E_{\max}$ model – THP-1 cells
3. Figure S3: compensation of hook effect through inhibition – two internal compounds
4. Table S1: input k_{cat} model – binding affinities ($K_{\mathrm{D,P}}$, $K_{\mathrm{D,E}}$ and α)
5. Table S2: input k_{cat} model – physiological parameters (P_0, E_0 and $t_{½,\mathrm{P}}$)

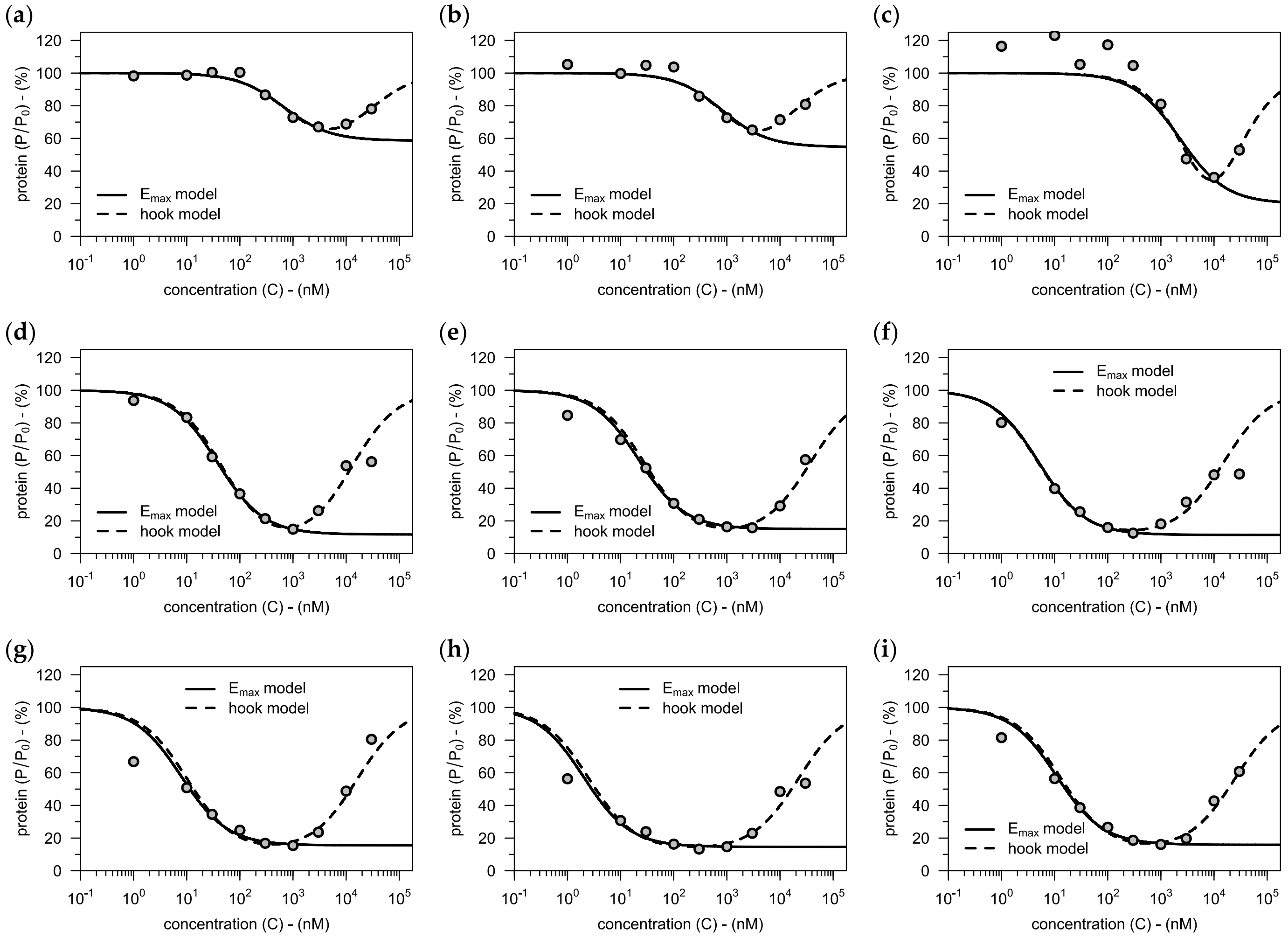

Figure S1. Relative levels of target protein are plotted against PROTAC concentration in media for the BTK degraders from Zorba et al. [1]. (**a-i**) The concentration-degradation profiles observed for *Cpds. A-I* in Ramos cells are fitted with the hook model and with the $E_{\max}$ model for comparison. When fitting the $E_{\max}$ model, only concentrations below the concentration of maximal degradation were considered.

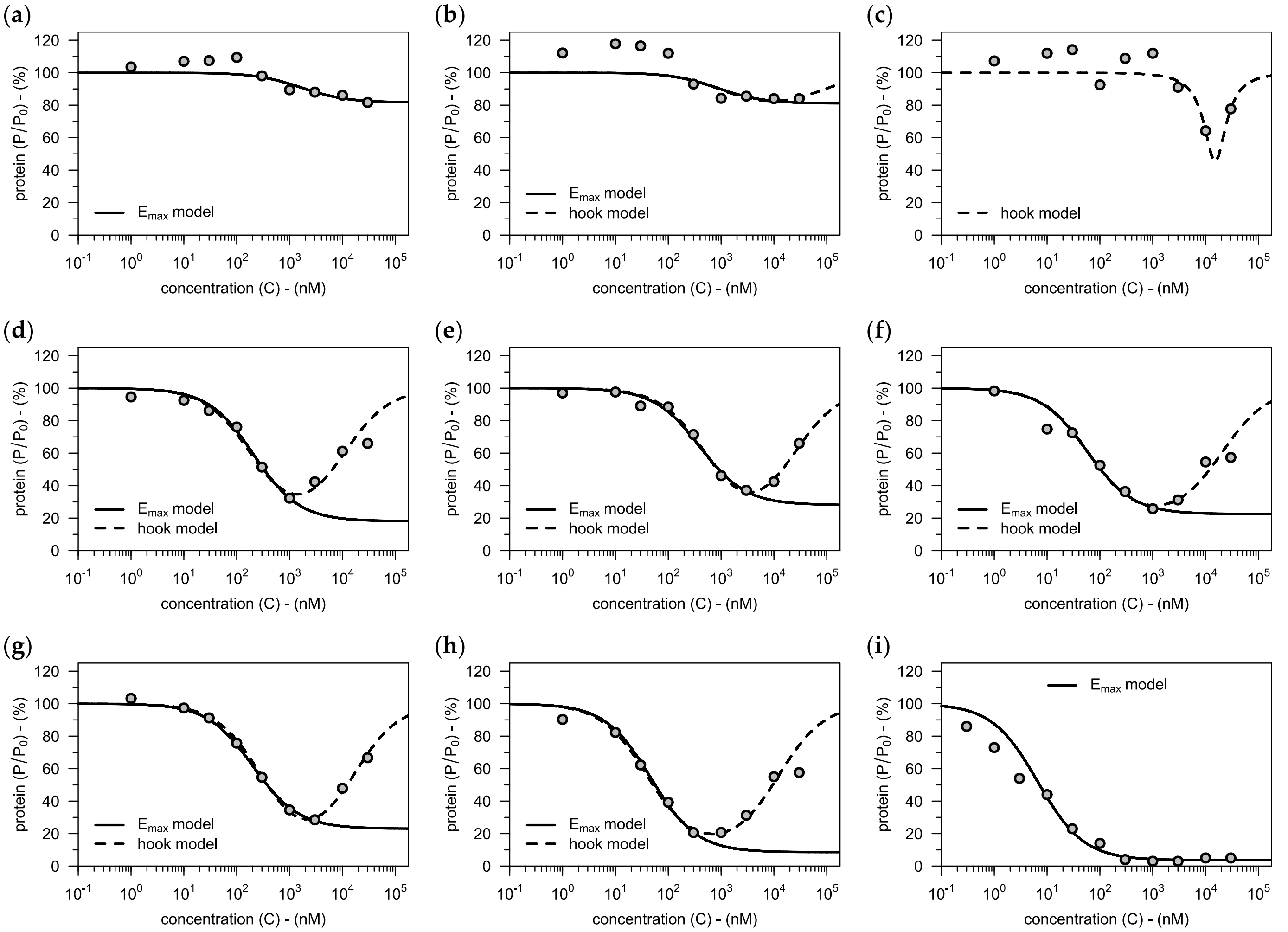

Figure S2. Relative levels of target protein are plotted against PROTAC concentration in media for the BTK degraders from Zorba et al. [1] (**a-h**) The concentration-degradation profiles observed for *Cpds. A-H* in THP-1 cells are fitted with the hook model and with the $E_{\max}$ model for comparison. When fitting the $E_{\max}$ model, only concentrations below the concentration of maximal degradation were considered. For the concentration-degradation profile of *Cpd. I* in THP-1 cells see Figure 2a. In the case of *Cpd. A*, only the $E_{\max}$ model is shown, as no hook effect is present in the data. In the case of *Cpd. C*, only the hook model is shown, as the $E_{\max}$ model did not converge. (**i**) The concentration-degradation profile observed for *Cpd. H* in rat splenocytes is described with the $E_{\max}$ model only, as no clear hook effect is present in the data.

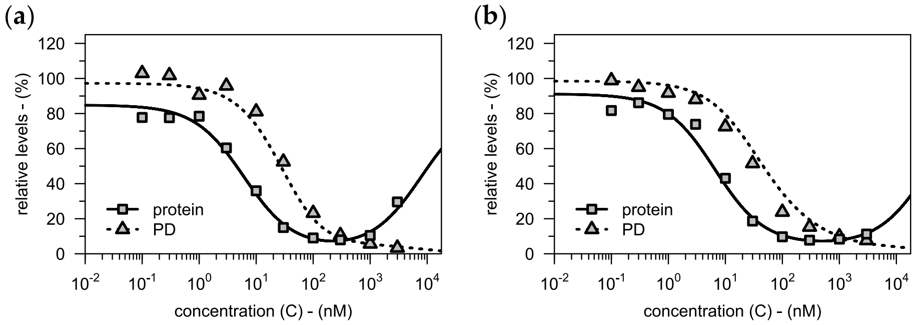

Figure S3. Target protein levels and the downstream pharmacodynamic response are plotted against drug concentration for two additional in-house PROTACs (one is shown in **a** and the other one in **b**). The hook model is used to assess degradation, which is then used to fit the PD model (fitted to all three (two here and one in Figure 5a) compounds simultaneously). As predicted by the model (see Appendix D for derivation), there is no hook effect present on the level of the downstream pharmacodynamic response.

Table S1. For each of the nine PROTAC compounds, the three binding affinity parameters reported by Zorba et al. [1] are stated. For the bimolecular dissociation constants, the values obtained by equilibrium mode analysis of the surface plasmon resonance data were used.

ID	$K_{D,P}$ (nM)	$K_{D,E}$ (nM)	α (1)
A	1535	15700	0.89
B	489	5300	0.47
C	1150	8800	2.50
D	71	2500	0.86
E	79	2700	0.83
F	80	3600	1.05
G	74	3200	1.21
H	61	3000	1.02
I	138	3100	1.34

Table S2. For each of the three cell types, the baseline concentrations of E3 ligase and protein of interest (i.e., BTK) are stated together with protein half-life. Baseline E3 ligase and target concentrations were calculated according to the total protein approach [2] from data reported by Zorba et al. [1]. BTK half-life had been reported by Bradshaw et al. for Ramos cells [3] and the same value was also applied to THP-1 cells. For rat splenocytes, the geometric mean of the half-lives reported by Mathieson et al. [4] for different human primary cells was used.

Cell Type	E_0 (nM)	P_0 (nM)	$t_{½,P}$ (h)
Ramos	203	1231	16
THP-1	120	1311	16
Rat Splenocytes	120	427	70

References

1. Zorba, A.; Nguyen, C.; Xu, Y.; Starr, J.; Borzilleri, K.; Smith, J.; Zhu, H.; Farley, K.A.; Ding, W.D.; Schiemer, J.; Feng, X.; Chang, J.S.; Uccello, D.P.; Young, J.A.; Garcia-Irrizary, C.N.; Czabaniuk, L.; Schuff, B.; Oliver, R.; Montgomery, J.; Hayward, M.M.; Coe, J.; Chen, J.; Niosi, M.; Luthra, S.; Shah, J.C.; El-Kattan, A.; Qiu, X.; West, G.M.; Noe, M.C.; Shanmugasundaram, V.; Gilbert, A.M.; Brown, M.F.; Calabrese, M.F. Delineating the Role of Cooperativity in the Design of Potent PROTACs for BTK. *Proc. Natl. Acad. Sci. USA* **2018**, *115*, E7285–E7292. https://doi.org/10.1073/pnas.1803662115
2. Guo, W.H.; Qi, X.; Yu, X.; Liu, Y.; Chung, C.I.; Bai, F.; Lin, X.; Lu, D.; Wang, L.; Chen, J.; Su, L.H.; Nomie, K.J.; Li, F.; Wang, M.C.; Shu, X.; Onuchic, J.N.; Woyach, J.A.; Wang, M.L.; Wang, J. Enhancing Intracellular Accumulation and Target Engagement of PROTACs with Reversible Covalent Chemistry. *Nat. Commun*. **2020**, *11*, 4268. https://doi.org/10.1038/s41467-020-17997-6
3. Bradshaw, J.M.; McFarland, J.M.; Paavilainen, V.O.; Bisconte, A.; Tam, D.; Phan, V.T.; Romanov, S.; Finkle, D.; Shu, J.; Patel, V.; Ton, T.; Li, X.; Loughhead, D.G.; Nunn, P.A.; Karr, D.E.; Gerritsen, M.E.; Funk, J.O.; Owens, T.D.; Verner, E.; Brameld, K.A.; Hill, R.J.; Goldstein, D.M.; Taunton, J. Prolonged and Tunable Residence Time Using Reversible Covalent Kinase Inhibitors. *Nat. Chem. Biol*. **2015**, *11*, 525–531. https://doi.org/10.1038/nchembio.1817
4. Mathieson, T.; Franken, H.; Kosinski, J.; Kurzawa, N.; Zinn, N.; Sweetman, G.; Poeckel, D.; Ratnu, V.S.; Schramm, M.; Becher, I.; Steidel, M.; Noh, K.M.; Bergamini, G.; Beck, M.; Bantscheff, M.; Savitski, M.M. Systematic Analysis of Protein Turnover in Primary Cells. *Nat. Commun*. **2018**, *9*, 689. https://doi.org/10.1038/s41467-018-03106-1
